# Usefulness of intraductal RFA in patients with malignant biliary obstruction

**DOI:** 10.1097/MD.0000000000021724

**Published:** 2020-08-14

**Authors:** Sung Yong Han, Dong Uk Kim, Dae Hwan Kang, Dong Hoon Baek, Tae Hoon Lee, Jae Hee Cho

**Affiliations:** a Department of Internal Medicine and Biomedical Research Institute, Pusan National University Hospital, Busan; b Department of Internal Medicine and Biomedical Research Institute, Pusan National University Hospital, Pusan National University School of Medicine, Busan, South Korea; c Department of Internal Medicine, Medical Research Institute, Pusan National University School of Medicine and Research Institute for Convergence of Biomedical Science and Technology, Pusan National University Yangsan Hospital, Yangsan-si, Gyengsangnam-do; d Digestive Disease Center and Research Institute, Department of Internal Medicine, SoonChunHyang University School of Medicine, Cheonan; e Department of Internal Medicine, Gangnam Severance Hospital, Yonsei University College of Medicine, Seoul, South Korea.

**Keywords:** biliary tract cancer, ERCP, radiofrequency ablation, perihilar cholangiocarcinoma, self-expandable metallic stents

## Abstract

**Background/Aims:**

Intraductal radiofrequency ablation (ID-RFA) is a novel therapy for unresectable malignant biliary obstructions. ID-RFA for perihilar lesions is associated with a high risk of adverse events. We aimed to evaluate the feasibility and efficacy of temperature-controlled ID-RFA for perihilar malignant biliary obstruction.

**Methods:**

Sixteen patients with pathologically proven perihilar cholangiocarcinoma were prospectively enrolled to evaluate the feasibility of hilar ID-RFA. Clinical efficacy and outcomes were subsequently evaluated in a multicenter retrospective cohort.

**Results:**

Nine of the 16 patients in the prospective cohort had Bismuth type IV and 7 had type IIIA perihilar cholangiocarcinoma. The median length of stricture was 34.5 mm. The median number of ID-RFA sessions was three, and all sessions were technically and functionally successful without severe adverse events. Clinical outcomes were assessed using a multicenter hilar ID-RFA cohort of 21 patients; the median stent patency and overall survival were 90 days (range: 35–483 days) and 147 days (range: 92–487 days), respectively. An approximate 16-month patency of the bile duct was maintained in one patient who had an intraductal growth pattern. In a comparison of the self-expandable metallic stent (SEMS) and plastic stent (PS) after hilar ID-RFA, no differences in stent patency (89 vs 90.5 days, respectively; *P* = .912) and adverse events (20.0% vs 10%, respectively; *P* = .739) were observed.

**Conclusions:**

ID-RFA at 7 W for 120 seconds is safe and feasible in patients with advanced perihilar cholangiocarcinoma. After ID-RFA, SEMS and PS placement showed comparable patency and survival rates.

**Trial registration number:**

KCT0003223

## 1 Introduction

Local ablation therapy is performed to maintain ductal patency, which improves survival and quality of life in patients with unresectable hilar cholangiocarcinoma. The endoscopic deployment of a self-expandable metallic stent (SEMS) is the standard palliation in these patients, and a SEMS has a life expectancy over 3 months, providing a high efficacy of bile drainage, low incidence of complications, and patient acceptance.^[1]^ However, the efficient drainage of hilar biliary strictures is challenging because of long and tight strictures, acute angulation of the bile duct, and complicated structures, such as the portal vein. The successful biliary drainage remains controversial especially as regards the use of a SEMS or plastic stent (PS). Both stent types completely occlude within 6 months despite improvements in anticancer treatments for perihilar cholangiocarcinoma; therefore, the development of a novel treatment method to prolong stent patency is necessary.

Newer endoscopic palliative therapies, including local ablative techniques, have been introduced. Photodynamic therapy has been reported to be an effective adjunctive therapy in unresectable cholangiocarcinoma.^[2]^ However, photodynamic therapy requires specialized facilities, has uncertain survival benefits, and can have complications, such as photosensitivity.^[3]^ Intraductal radiofrequency ablation (ID-RFA) using an endobiliary radiofrequency ablation (RFA) catheter has been identified as another treatment option. ID-RFA is easily performed on distal malignant biliary obstructions, and the local tumor is successfully ablated with minimal complications. However, ID-RFA in perihilar applications has the potential to cause bile duct perforation and vascular injuries, such as hemobilia and liver infarction, because the hilar bile duct is located near the hepatic artery and portal vein complexes.^[4–6]^ We aimed primarily to evaluate the feasibility and clinical outcomes of temperature-controlled hilar ID-RFA in patients with perihilar malignant biliary obstruction. Secondly, we sought to compare the clinical efficacy of a bilateral SEMS and PS after hilar ID-RFA.

## 2 Materials and methods

### 2.1 Study design and population

**Figure 1:**
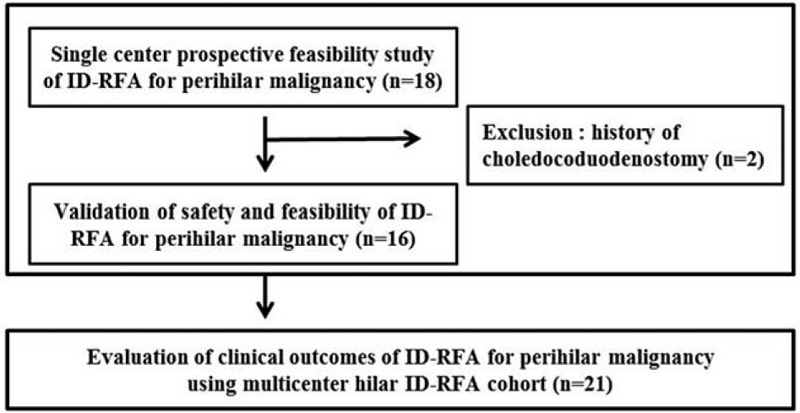
Flowchart of the study.

We performed a prospective, uncontrolled, single-center study that included 18 patients with unresectable perihilar cholangiocarcinoma. Two patients had a choledochoduodenostomy and were excluded from the analysis. The inclusion criterion was pathologically confirmed perihilar cholangiocarcinoma of Bismuth type III or IV. The decision of whether a patient had an unresectable malignancy was based on their medical fitness or disease extent (tumor extending into both liver lobes, major vascular involvement, or Bismuth type IV). The ethics committee of the institutional review board of the hospital approved this prospective study (H-1401-005-013), which was registered in the Clinical Research Information Service (KCT0003223). After a feasibility evaluation, a further retrospective analysis of clinical outcomes regarding hilar ID-RFA was conducted using a multicenter hilar ID-RFA cohort of 21 patients (an additional 5 patients underwent hilar ID-RFA at 7 W and 120 seconds from Gil Medical Center) (Fig. 1). The baseline characteristics of patients from each hospital were not statistically different between the two groups (data not shown).

### 2.2 Clinical trial protocol: ID-RFA procedure

All patients underwent endoscopic retrograde cholangiopancreatography (ERCP) before enrollment for tissue confirmation and biliary drainage. ID-RFA was subsequently performed using a newly designed ID-RFA catheter (ELRA STARmed, Seoul, Korea) and a transpapillary approach. The ID-RFA catheter is a single-use, disposable, bipolar device that is suitable for endoluminal delivery of RFA into the biliary tree over a guidewire. In addition, the catheter can be deployed through a side-view endoscope with 4.2-mm diameter working channels (Olympus TJF-260 V, Tokyo, Japan) or a forward-view endoscope with 3.8-mm diameter working channels (Olympus GIF-260, Tokyo, Japan). Each ID-RFA session was applied at 7 W (11 or 18 mm) for 120 s under an 80°C intraductal target temperature. During the procedure, the wattage was automatically guided by a STARmed RF generator (STARmed, Seoul, Korea). The prevention of tissue charring is the major advantage of temperature-controlled ID-RFA; this may reduce the risk of ID-RFA induced perforation and bleeding. The actual ID-RFA procedure is as follows: The ID-RFA catheter is placed within the tumor stricture under fluoroscopic visualization of the biliary system, and repeated applications of ID-RFA are carefully made by passing the short-length ID-RFA catheter (11 or 18 mm) to the treated segments (1–5 applications per intervention); after ID-RFA treatment, either a bilateral Y-configured SEMS (Niti-S Biliary Y or T-stent, Taewoong, Goyang, Korea; Niti-S or Niti-D stent, Taewoong Medical, Goyang, Korea), bilateral uncovered SEMS (Benefit stent, MiTec, Seoul, Korea), or bilateral PS (Boston scientific, Boston, MA, USA & Cook medical, Bloomington, IN, USA) is placed depending on the clinical condition of each patient.

### 2.3 Outcome measurement parameters

We evaluated adverse events and biliary patency. The outcome of bilateral drainage was evaluated according to the following parameters:

1) Technical success was defined as the proper placement of the ID-RFA catheter in the stricture site and successful ablation, including a successful bilateral SEMS or PS placement across the stricture.
2) Functional success was defined as radiographically adequate positioning, immediate biliary decompression, and at least a 30% reduction of serum bilirubin level within 2 weeks.
3) Procedure-related adverse events and mortality.
4) Stent patency (the period between stent insertion and occlusion or patient's death). Occlusion of the stent was confirmed if patients had recurrent or persistent jaundice with cholestasis or cholangitis (leukocytosis, fever, increasing bilirubin).

### 2.4 Statistical analysis

Statistical analysis was performed using SPSS software (version 22.0, IBM Corp., Armonk, NY). Categorical data were expressed as frequencies and percentages, and between-group differences were evaluated using the Fisher Exact test. Continuous data were expressed as medians with ranges, and between-group differences were evaluated using the Mann-Whitney *U* test. Statistical significance was set at *P* < .05. For comparison, stent patency was assessed using Kaplan–Meier survival curves and the log-rank test.

## 3 Results

### 3.1 Patient characteristics and clinical outcomes of ID-RFA in the prospective feasibility cohort

Figure 1 shows the flowchart of this study. Detailed information on the patient characteristics and results of the ID-RFA procedure in the prospective cohort is shown in Table 1. The 16 enrolled patients included 6 women (median age: 75.5 years; range: 59–85 years). Ten patients had hypertension, 4 patients had diabetes mellitus, and no patients had liver cirrhosis or viral hepatitis. Of the 16 enrolled patients, 9 had Bismuth type IV and 7 had type IIIA perihilar cholangiocarcinoma. The median stricture length was 34.5 mm (range: 17–63 mm). The median number of ID-RFA applications per single session was 3 (range: 1–5). ID-RFA was performed in 10 patients using an 11-mm RFA catheter (62.5%), in 7 patients using an 18-mm catheter, and in 1 patient using both catheters.

**Table 1:**
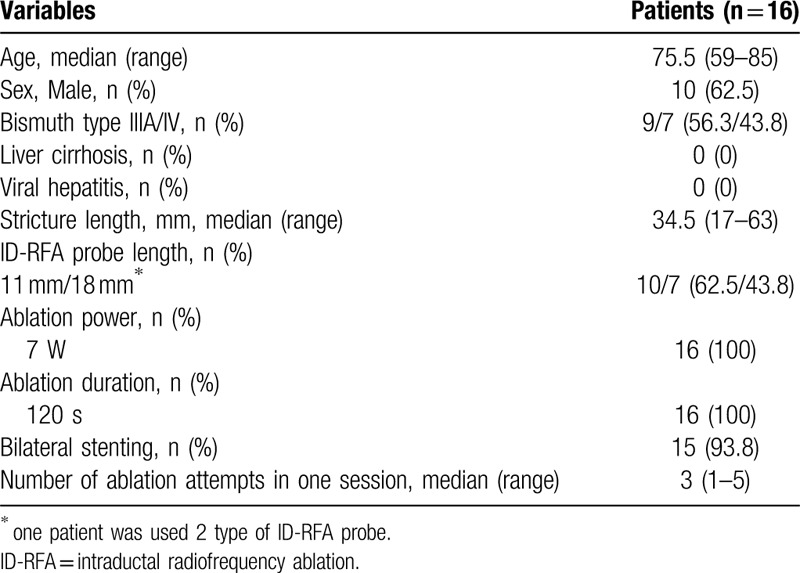
Baseline characteristics and result of ID-RFA procedure.

The clinical outcomes of the ID-RFA procedure in the prospective cohort are shown in Table 2. The median initial total bilirubin level was 10.39 mg/dL. In all patients, ID-RFA was technically and functionally successful. After ID-RFA, 6 patients had placement of a bilateral SEMS, 9 patients had placement of a bilateral PS, and 1 patient had insertion of an endoscopic nasobiliary drainage (ENBD) tube. The median total bilirubin level was 2.18 mg/dL at 1 day after ID-RFA and 1.30 mg/dL at 2 weeks after ID-RFA. No fatal adverse events, such as major bleeding or perforation, were observed. The median duration of stent patency was 91.5 days (35–483 days). The median overall survival period was 131 days (50–487 days). No difference in stent patency between the bilateral SEMS and PS groups (93.5 vs 91 days, respectively, *P* = .607) was observed. Interestingly, 1 patient who had an intraductal growth pattern was eligible for repeated ID-RFA and survived for 332 days without internal or external biliary drainage.

**Table 2:**
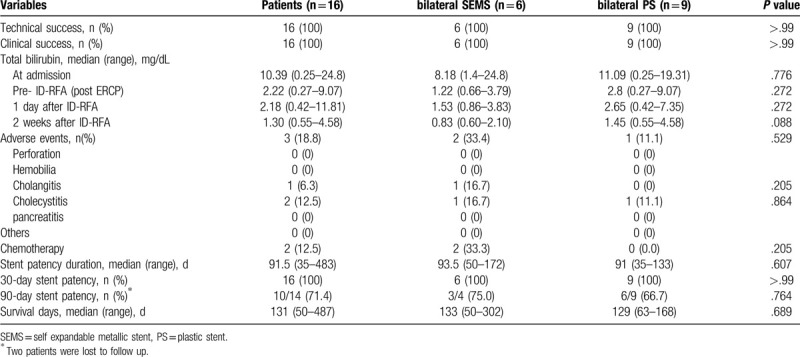
Clinical outcomes of ID-RFA in prospective feasibility cohort.

### 3.2 Clinical outcomes in the multicenter hilar ID-RFA cohort

Table 3 shows the clinical outcomes and baseline characteristics of patients in the multicenter hilar RFA cohort. The baseline characteristics did not differ significantly between the SEMS and PS groups. The median stricture length was 34 mm (range: 17–63 mm). The median number of ID-RFA applications per single session was 3 (range: 1–5). ID-RFA was performed in 15 patients using an 11-mm RFA catheter (71.4%), in 7 patients using an 18-mm catheter, and in 1 patient using both catheters. The median duration of stent patency was 90 days (35–483 days), and no difference was observed in the stent patency between the SEMS and PS groups following ID-RFA (median stent patency: 89 vs 90.5 days, respectively; *P* = .912). The functional duration of the stent was not successfully identified in 2 patients; therefore, the last follow-up visit was considered as the stent occlusion. The median overall survival period was 147 days (50–487 days); the SEMS group showed slightly longer overall survival than the PS group, but the difference was not statistically significant (median overall survival: 157 vs 119.5 days; *P* = .142). Only 2 patients in the SEMS group received chemotherapy.

**Table 3:**
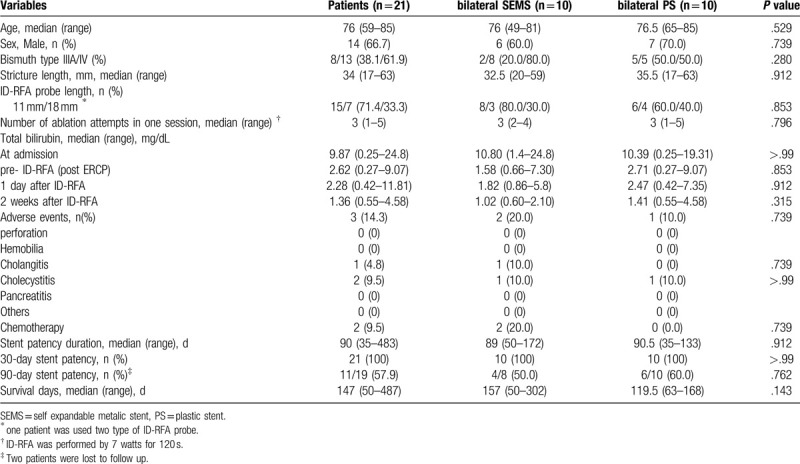
Clinical outcome of multicenter hilar RFA cohort.

**Figure 2:**
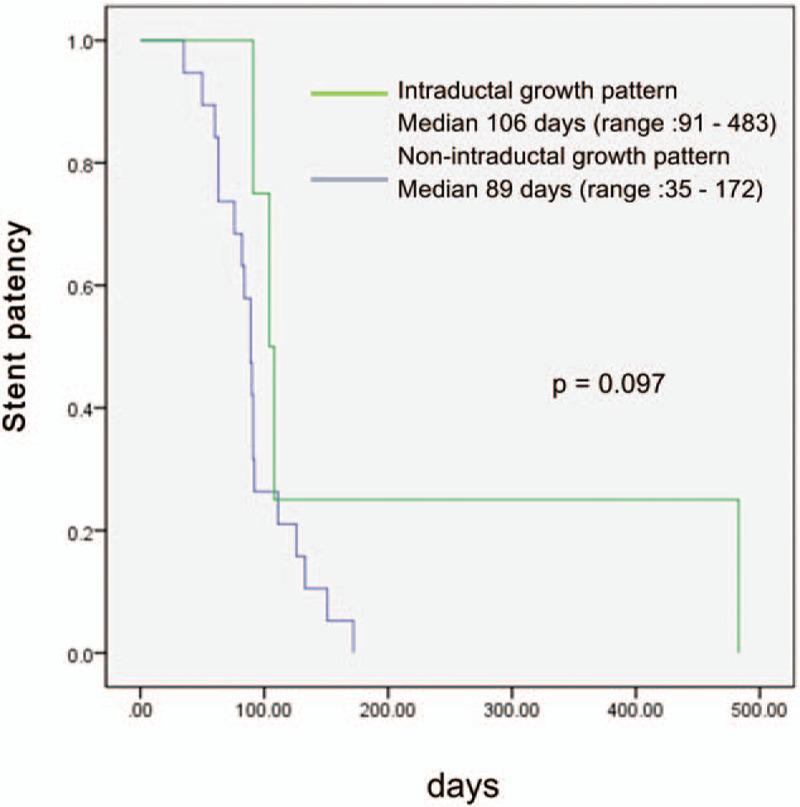
Kaplan-Meier curve for stent patency according to whether or not of intraductal growth pattern.

One patient with cholangitis was controlled with intravenous antibiotics, and 2 patients with cholecystitis underwent percutaneous cholecystostomy. In both the SEMS and PS groups, no significant difference in the adverse event rate was observed (20% vs 10%, respectively, *P* = .739). Interestingly, the ENBD tube was removed in one patient with an intraductal growth pattern, and drainage was possible for a long period without any additional procedure (483 days). Figure 2 shows the result of stent patency according to intraductal growth pattern, and no significant difference between the two groups was observed. However, the patients with an intraductal growth pattern tended to have longer stent patency than other patients (106 vs 89 days, *P* = .097).

## 4 Discussion

Temperature-controlled ID-RFA was safe and feasible in patients with advanced hilar cholangiocarcinoma and had no major adverse events, such as perforation and hemobilia. For the hilar ID-RFA, we used a short ID-RFA catheter under a low power (7 W) for a duration of 120 seconds. Because stricture length has a wide range (17–63 mm), we performed multiple overlapping ID-RFAs to cover the entirety of perihilar strictures. RFA can also be applied to more than one stricture site during the same session, such as the common hepatic duct or left- or right-hepatic duct, in patients with Klatskin tumors. In the prospective feasibility study, technical and clinical success was achieved in all enrolled patients, and our adverse event rate of 18.8% is comparable to that of other studies (0–33.3%).^[7–10]^

No specific recommendation has been given for the ID-RFA settings of duration, energy, and technique for hilar applications. The actual ID-RFA ablation depth in the human bile duct is approximately 4 mm (1.7–4.3 mm), and the effective length of bile duct ablation has been estimated to be 40% to 72% of the length of the RFA catheter.^[11–13]^ A previous in vivo swine study showed that hilar ID-RFA related perforation in the normal bile duct occurred when a duration of over 90 seconds was used.^[7]^ However, some clinical studies of hilar ID-RFA revealed no perforation at a duration up to 120 seconds.^[9,11]^ Since a cancerous bile duct has a thicker wall than a normal bile duct, we chose to perform hilar ID-RFA for a duration up to 2 minutes to get better outcomes. We chose shorter ID-RFA catheters because longer catheters are not suitable for intraductal ablation, since the perihilar bile duct is angulated and separated, and longer catheters have the potential to cause bilo-vascular complications. Therefore, we recommended the use of low power and short ID-RFA catheters (7 W and 11 mm or 18 mm) for the safe and successful performance of hilar ID-RFA. When dealing with long segments of bile duct cancer, repeated ID-RFA with a tandem overlapping of RFA catheters can overcome the limitations of ID-RFA.^[7]^ Furthermore, since ERCP is not always successful when a perihilar biliary obstruction is present, the percutaneous transhepatic biliary drainage (PTBD) tract can be used as an alternative to ERCP-guided RFA.^[7–10]^ Consequently, the utilization of a multidisciplinary care approach through the effective application of ERCP and PTBD is necessary in patients with a perihilar biliary obstruction.

The multicenter hilar ID-RFA cohort had a median stent patency of 90 days, and survival was 147 days; these results are similar to those of a Klatskin tumor without hilar ID-RFA. Therefore, we could not confirm whether the hilar RFA was effective for these patients. Most patients in this study were in poor health, which made additional chemotherapy impossible. Therefore, we admit that the actual efficacy of hilar ID-RFA could not be verified in this study. In addition, we compared the clinical usefulness of a PS to a SEMS after hilar ID-RFA because insufficient data is available on which stent to use after hilar ID-RFA. Our study shows no difference in stent patency, survival, and adverse events between a PS and SEMS. In our study, a SEMS did not show a superior effect compared to a PS in hilar malignancy, unlike other studies, and the replaceable PS allows for repeated RFA, which is associated with a survival benefit.^[14]^ A PS may be a more reasonable therapeutic option after hilar ID-RFA; however, this should be confirmed through further studies because the selection of stent type depends on the patient's condition.

Determining a subgroup that can expect a therapeutic effect after hilar ID-RFA is very important when deciding whether to perform hilar ID-RFA. Notably, an approximate one-year patency of the bile duct was achieved in one patient who had an intraductal growth pattern of bile duct cancer; this patient did not receive any additional therapy, including chemotherapy. The intraductal growth pattern cholangiocarcinoma constitutes 8% to 18% of cholangiocarcinomas.^[15–17]^ In our study, a total of 4 patients had cholangiocarcinoma with an intraductal growth pattern. We observed a tendency to maintain longer stent patency in the patients with an intraductal growth pattern than that in the patients without an intraductal growth pattern, but the difference was not statistically significant. Therefore, cholangiocarcinoma with an intraductal growth pattern may show a better response to RFA treatment. One patient was able to maintain stent patency for 1 year through repeated ID-RFA; such repeated RFA is thought to help extend the total duration of stent patency. Further research is required to determine whether patients with repeated RFA and an intraductal growth pattern have a good prognosis.

Our research has several limitations. First, we enrolled a small number of patients who were treated in a single institution in the prospective feasibility cohort and did not have a control group. Further randomized, controlled studies are needed to prove the usefulness of ID-RFA for perihilar cholangiocarcinoma. Second, patients with advanced diseases were predominantly enrolled, and the proportion of patients treated with chemotherapy was small. In particular, the advanced stages were analyzed heterogeneously, including locally advanced stages and distant metastases. Furthermore, the SEMS group had a heterogeneous mix of procedures, such as stent-in-stent or stent-by-stent, and various stent diameters were used (8 mm/10 mm). Finally, the proportion of patients for whom follow-up was discontinued was high (33.3%), and this affected the accuracy of the assessment.

In conclusion, ID-RFA at 7 W for 120 seconds is safe and feasible for advanced hilar obstruction. After ID-RFA, both SEMS and PS placement showed comparable patency and survival rates. Cholangiocarcinoma with an intraductal growth type and repeated ID-RFA is thought to be a way to maximize the effect of ID-RFA. For repeated ID-RFA, a PS after ID-RFA may be beneficial. Long-term survival gains could be achieved if these local therapies are used appropriately. However, randomized, controlled studies and multi-institutional studies, especially ones that compare RFA with a control, need to be conducted to confirm the benefits of long-term stent patency and patient survival.

## Author Contribution

**Critical revision:** Jae Hee Cho, Tae Hoon Lee.

**Design of the work:** Dong Uk Kim, Dae Hwan Kang.

**Drafting the article:** Sung Yong Han.

**Data acquisition & analysis:** Sung Yong Han, Dong Hoon Baek.
